# Anatomical variations and abnormalities of the maxillary region and clinical implications: A systematic review and metaanalysis

**DOI:** 10.1097/MD.0000000000034510

**Published:** 2023-09-22

**Authors:** Juan José Valenzuela-Fuenzalida, Belén Baez-Flores, Roberto Ávila Sepúlveda, Claudia Moya Medina, Rubén Pérez, Esteban López, Juan Sanchis, Mathias Orellana Donoso, Javiera Leyton Silva, Macarena Cecilia Rodriguez, Joe Iwanaga

**Affiliations:** a Departamento de Morfología, Facultad de Medicina, Universidad Andres Bello, Santiago, Chile; b Departamento de Ciencias Química y Biológicas Facultad de Ciencias de la Salud, Universidad Bernardo O’Higgins, Santiago, Chile; c Department of Morphology and Function, Faculty of Health and Social Sciences, Universidad de las Américas, Santiago, Chile; d Giaval Research Group, Faculty of Medicine, University of Valencia, Valencia, Spain; e Escuela de Medicina, Universidad Finis Terrae, Santiago, Chile; f Department of Neurosurgery, Tulane Center for Clinical Neurosciences, Tulane University School of Medicine, New Orleans, LA.

**Keywords:** anatomical variations, anomalies, clinical anatomy, maxilary bone, maxillary sinus, paranasal sinus, sinusitis

## Abstract

**Objective:**

The objective of this review is to investigate and analyze the anatomical variations present in the maxillary sinus (MS), through the examination of the prevalence of these variations, as well as the corresponding prevalence of clinically significant pathologies and complications associated with them.

**Methods:**

The search process was carried out in the following databases; MEDLINE, SCIELO, WOS, CINHAL, SCOPUS, and GOOGLE SCHOLAR, using as search terms; “Maxillary bone,” “Maxillary sinus,” “Paranasal sinus,” “Anatomical variations,” “Sinusitis” and “Clinical anatomy.”

**Results:**

A total of 26 articles and 12969 samples were included, from which 12,594 subjects had their sex recorded giving a total of 5802 males and 6792 females. The variants reported by the included were Haller cells, Concha Bullosa, Number of septa, Hypoplastic sinus, Agger Nasi, Thickening of the MS mucosa, Deviation of the nasal septum, Accessory ostium, and Onodi cells. Among the mentioned, the ones that presented the greatest number of studies (between 8 and 10 studies included) were: the Haller Cells, the Concha Bullosa, and the Number of septa, where prevalence was 0.30, 0.36, 0.39 respectively. These variations can lead to sinusitis, cause some types of tumors, or affect neighboring structures that could be compromised by this variation.

**Conclusion:**

As a result, it is certainly complex to distinguish the presence of anatomical variations from pathological abnormalities. Therefore, knowledge of the different variations and their clinical relationships could be a useful asset for clinicians dedicated to this region.

## 1. Introduction

**Figure 1.**
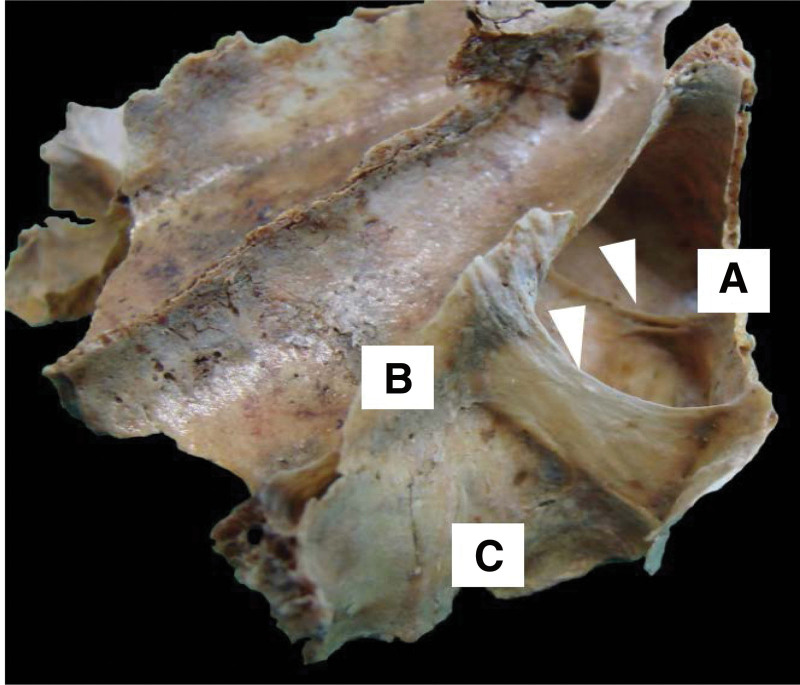
Osteological structure showing the left MS. The anterior wall (A) is observed; inner wall or base (B), and the posterior wall (C), the arrows indicate 2 transverse septa. The black arrow indicates a channel on one of the septa for the passage of vessels and nerves. MS = maxillary sinus.

**Figure 2.**
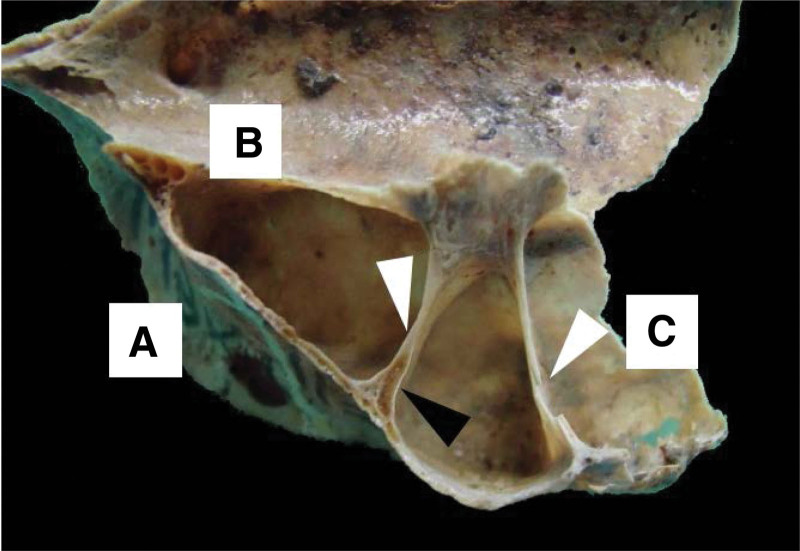
Osteological structure showing the left MS. The anterior wall (A) is observed; inner wall or base (B), and the posterior wall (C), the arrows indicate 2 transverse septa. The black arrow indicates a channel on one of the septa for the passage of vessels and nerves. MS = maxillary sinus.

**Figure 3.**
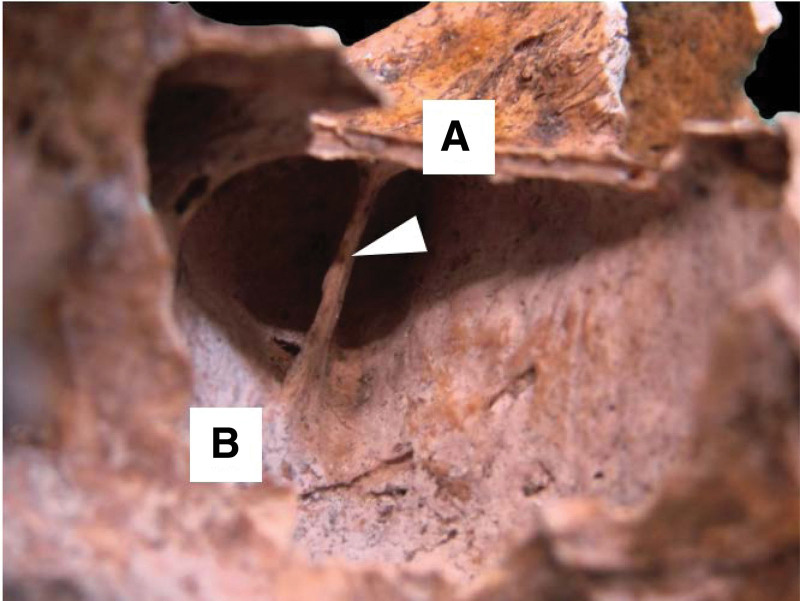
Osteological structure showing the right MS. The upper wall (A) is observed; lower wall or floor (B), the arrow indicates an oblique septum that extends to the upper wall of the MS, dividing it into a medial and a lateral compartment. MS = maxillary sinus.

**Figure 4.**
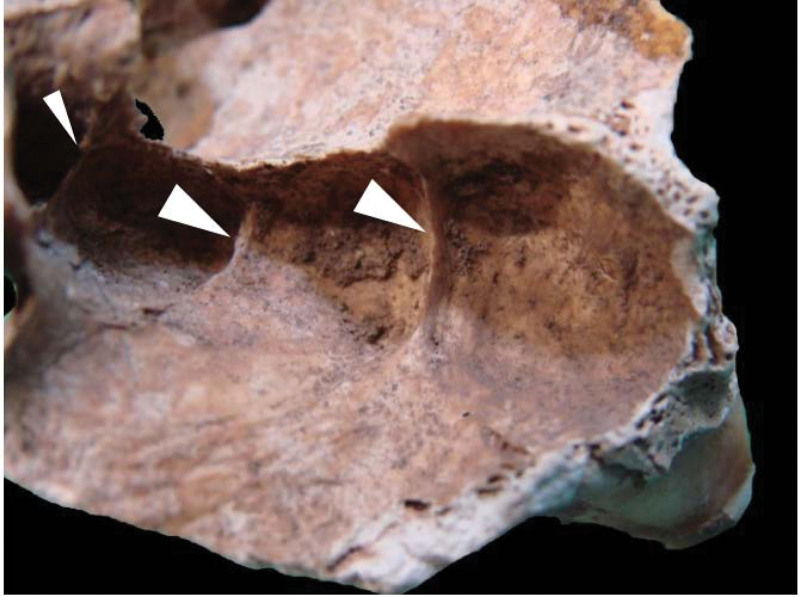
Osteological specimen showing the left MS. Arrows indicate 2 underdeveloped transverse septa that divide the floor into 4 compartments. MS = maxillary sinus.

**Figure 5.**
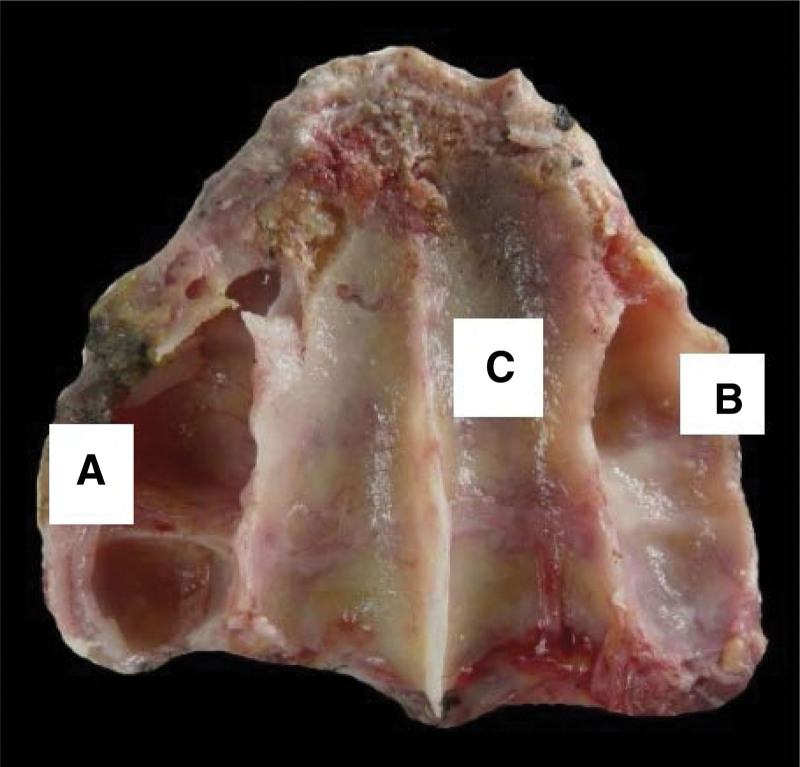
Structure of the dissection of the MB, it is observed in superior view to the left MS (A) and right (B) and the floor of the nostrils (C). MB = maxillay bone, MS = maxillary sinus.

**Figure 6.**
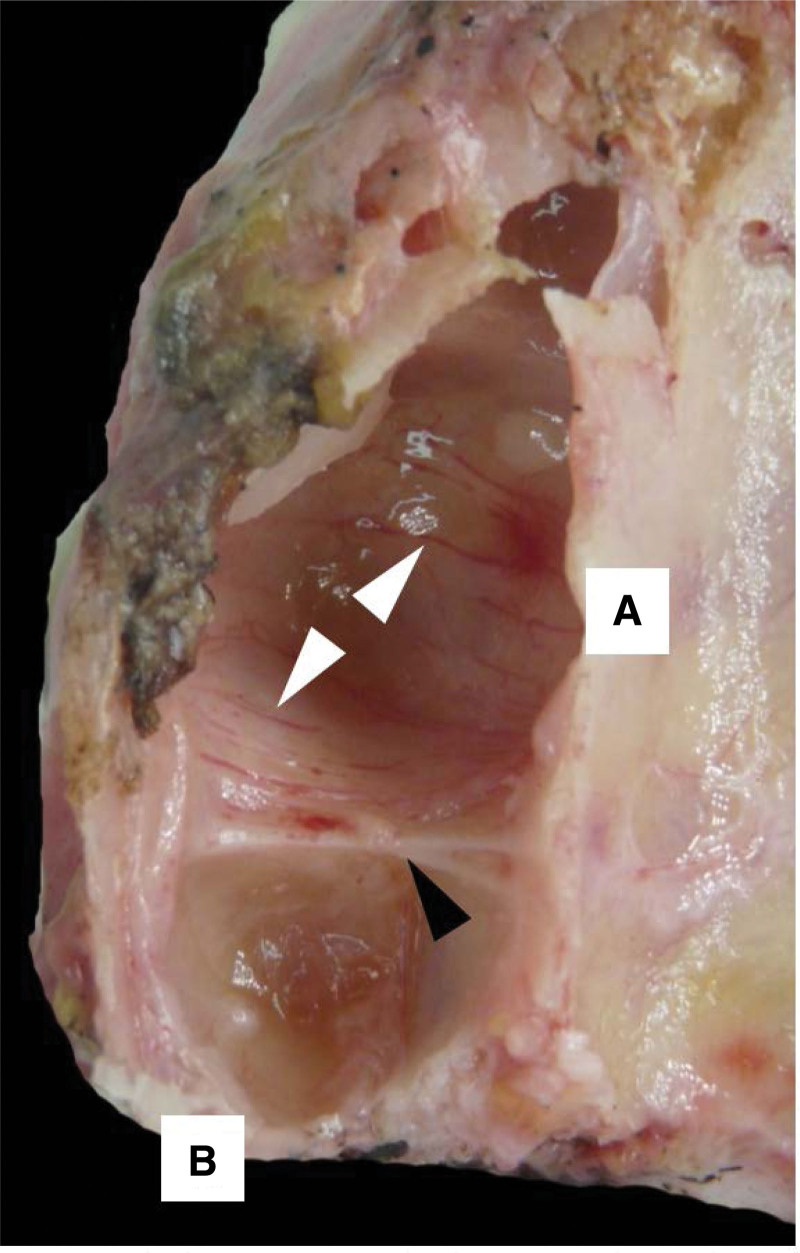
Dissection piece showing the left MS. The inner wall or base (A), the posterior wall or tuberosity (B) can be seen, the black arrow indicates a transverse septum, and the white arrows indicate arterial vessels that make up the irrigation system of the antral mucosa. MS = maxillary sinus.

Anatomically, the maxillary sinus (MS) is a complex structure to study, due to its walls and communication with different structures, such as the respiratory system, upper teeth, and the orbital cavity, specifically the floor and the inferomedial region of this cavity. The MS is a pneumatic cavity, housed within the body of the Maxilla, that communicates with the nasal cavity through the Ostium, at the middle nasal meatus. In the literature, the description of the MS uses some eponyms and synonyms that should be considered, these include: Geniantrum, Highmore cave, Highmore antrum, and Infraorbital recess. In addition, it is necessary to know the communications that it presents with the cells of the Ethmoid bone, the lower wall of the orbital cavity, the upper part of the shell nasal media, the anterior wall of the Sphenoid sinus, the alveolar processes of the Maxilla, and other structures. The MS varies in architecture, dimensions, and morphometry between individuals, and although the right and left MS tend to be symmetrical, slight differences in laterality may be present.^[1]^ Figures 1 to 6 will characterize the MS and its walls. (Figs. 1–6).

Morphologically, the MS is studied as a triangular pyramid when its lower edge is not considered a surface. When this edge reaches a prominent expansion, it can be considered a true wall, acquiring the shape of a quadrangular pyramid whose base is medial and parallel to the lateral wall, while the apex is facing the palatal process of the Maxillary bone (MB). *The anterior wall is convex and corresponds to the Canine Fossa, whose concavity stands out towards the opening of the MS, above the Infraorbital canal. The antero-superior and middle alveolar canals run through the anterior and anterolateral walls, respectively. The upper, or orbital, wall corresponds to the floor of the orbit, which slopes slightly laterally and inferiorly, and where the infraorbital canal appears as an elongated eminence from anterior to posterior.* The posterior, or Pterygomaxillary wall, corresponds to the Zygomatic Fossa and is related to the tuberosity of the Maxillary. The Intersinusonasal Septum, or nasal wall, is formed by a part of the lateral wall of the Nasal Fossae (Figs. 1–6).^[2,3]^ The less vertical bone availability found in the posterior Maxilla could be caused by excessive pneumatization of the sinus, increased resorption of the edentulous ridge, or a combination of both. Among the causes of increased reabsorption of the toothless ridge, we have the duration of edentulism; frequency, direction, and intensity of forces acting against the alveolar process; adjustment of a previously carried prosthesis; advanced periodontal disease; and systemic factors such as age, sex, hormonal disorders, metabolic factors, and inflammation.^[4–8]^

Along the nasal passages, the Paranasal Sinuses constitute a unique anatomical, functional, pathological, and therapeutic complex.^[9]^ They participate in all the functions of the nasal passages, except for the smell.^[10]^ Although a wide variety of primary functions have been assigned to the paranasal sinuses, none of the proposed ones have been universally accepted as an essential reason for their existence.^[11]^ Theories about the function of the MS, one of the Paranasal Sinuses, are diverse and all of them have their foundation and interest. As they are hollow cavities they contribute to the decrease in the weight of the skull.^[12]^ They also have a respiratory function, acting as an air reservoir for the correct functioning of the Naso-laryngeal-tracheo-bronchopulmonary apparatus by heating, humidifying, and purifying the respiratory mucosa.^[13]^ Additionally, it has a defensive, enzymatic, and protective function against bacterial and viral invasions because of the secretion mantle of the mucosa. A vocal function is also described, as the MS acts as a suitable resonance box, designed to sustain and amplify the emission of laryngeal sound, particularly during singing. Because of this, the characteristics of the voice are influenced by the state of the Paranasal Cavities, especially of the MS.^[14]^

Like any of the other Paranasal Sinuses, the MS can present anatomical variations including unilateral or bilateral alveolar pneumatization, hypoplasia, agenesis, antral septum, and exostosis. The MS can also present pathologies, for example, opacity, occupancy, mucosal retention pseudocysts, polyps, anthrocytes, and thickening of the mucosa. Knowing the possible anatomical variations that could be found within the MS, helps clinicians when making decisions regarding diagnosis, prognosis, and the development of a treatment plan for their patients.^[15–18]^ Extensive reports of the MS suggest that various clinical complications of the surrounding structures may be associated with the MS. For example, ample evidence, found in recent scientific literature, shows that dental sepsis commonly results in reactive mucosal thickening on the lower face of the MS.^[19]^ The hyperneumatization of the Maxillary Alveolar Processes is an odontogenic cause of maxillary sinusitis, a disease that affects the anterior Paranasal Sinuses, that is currently increasing in incidence, being more common than it had been previously thought.^[20]^ Opacification and sinusitis in the MS, which affects a group of the unilateral anterior sinus, in 75% and 25% to 40% of cases, respectively, have a dental etiology. Understanding the role of MS in health and disease requires a thorough understanding of the physiology of the upper respiratory tract and also of the development and clinical and imaging anatomy of MS, including its relationship with adjacent structures such as the dentition, nose, and ethmoid and frontal sinuses.^[21,22]^

The primary objective of this review is to investigate and analyze the anatomical variations present in the MS, through the examination of the prevalence of these variations, as well as the corresponding prevalence of clinically significant pathologies and complications associated with them. By conducting this investigation, the review seeks to significantly advance our understanding of the complexities surrounding the MS, providing a comprehensive and up-to-date collection of information on these variations. Moreover, the review aims to serve as a resource for healthcare professionals, researchers, and clinicians, offering them specific and complete knowledge about the variations encountered in the MS. By consolidating this information, the review also strives to enhance interventions, treatment proposals, and overall patient care.

## 2. Methods

### 2.1. Protocol

This systematic review and meta-analysis was performed and reported according to the Preferred Reporting Items for Systematic Reviews and Meta-Analyses (PRISMA) statement. This revision has been checked in to the OSF repository with the following doi; https://doi.org/10.17605/OSF.IO/WKQ2D.

### 2.2. Electronic search

**Figure 7.**
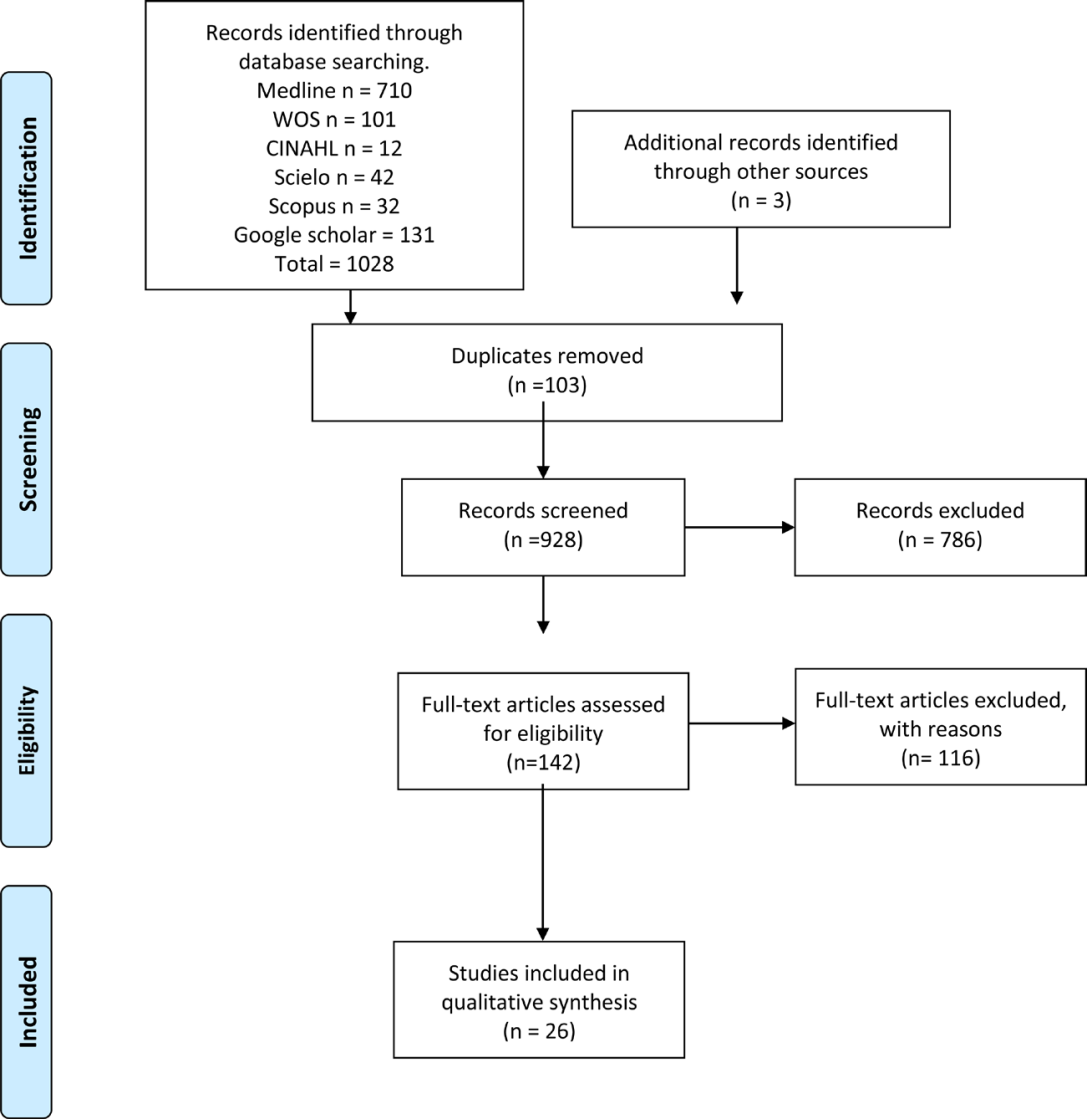
PRISMA flowchart diagram.

We systematically searched MEDLINE (via PubMed), Web of Science, Google Scholar, the Cumulative Index to Nursing and Allied Health Literature (CINAHL), Scopus, and the Latin American and the Caribbean Literature in Health Sciences (LILACS) from inception until may 2023 (Fig. 7). The search strategy included a combination of the following terms: “Maxillary bone” (No Mesh), “Maxillary sinus” (No Mesh), “anatomical variations” (No mesh) “Sinusitis “(Mesh term) “Paranasal sinus” (No Mesh), and “clinical anatomy” (No Mesh) using the Boolean connectors “AND,” “OR” and “NOT.” The search strategies for each database are available in the supplementary material (Table S1, Supplemental Digital Content, http://links.lww.com/MD/J382). Two authors (J.J.V. and C.M.) independently screened the titles and abstracts of the references retrieved from the searches. The full text for references, that either author considered to be potentially relevant, was obtained. A third reviewer (R.P.) was involved if consensus could not be reached.

### 2.3. Eligibility criterio

Studies on the presence of variants in the MS and their association with any clinical condition were considered eligible for inclusion if the following criteria were fulfilled: population: samples of dissections or images of the MS; outcomes: prevalence of the MS variants and their correlation with pathologies of the nasal cavity, MB pathologies, or dentistry complications. Additionally, anatomical variants were classified and described based on normal anatomy and classifications proposed in literature; studies: this systematic review included research articles, research reports, or original research published in English in peer-reviewed journals and that were indexed in some of the databases reviewed. Conversely, The exclusion criteria were as follows: population: animal studies, studies that performed variant analyzes of other sinuses, and studies: letters to the editor or comments.

### 2.4. Assessment of the methodological quality of the included studies

Quality assessment was performed using the methodological quality assurance tool for anatomical studies (AQUA) proposed by the International Evidence-Based Anatomy Working Group (IEBA) [Henry et al, 2015]. Data extraction and quality assessment were independently performed by 2 reviewers (J.J.V. and J.M.). We involved a third reviewer (A.B.) if a consensus could not be reached. The agreement rate between the reviewers was calculated using kappa statistics.^[23–25]^

### 2.5. Data collection process

Two authors (B.B. and E.L.) independently extracted data on the outcomes of each study. The following data were extracted from the original reports: authors and year of publication, country, type of study, sample characteristics (sample size, age, distribution, and sex), prevalence and morphological characteristics of MS, statistical data reported by each study, and main results.

### 2.6. Statistical methods

The data extracted in the meta-analysis were interpreted to calculate the prevalence of the MS variants using JAMOVI software. The jamovi project was founded to develop a free and open statistical platform which is intuitive to use and can provide the latest developments in statistical methodology. At the core of the jamovi philosophy, is that scientific software should be “community driven,” where anyone can develop and publish analyses, and make them available to a wide audience, it should be noted that JAMOVI is the name of the software and is not an abbreviation.^[26]^ The DerSimonian-Laird model with a Freeman-Tukey double arcsine transformation was used to combine the summary data. Due to the high heterogeneity in the prevalence data on MS variations, a random effects model was used. The degree of heterogeneity between included studies was assessed using the chi² test and the heterogeneity (I²) statistic. For the chi² test, a *P* value of less than 0.10, as proposed by the Cochrane Collaboration, was considered significant. Values of the I² statistic were interpreted as follows with a 95% confidence interval [CI]: 0% to 40% indicating no important heterogeneity, 30% to 60% indicating moderate heterogeneity, 50% to 90% indicating substantial heterogeneity, and 75% to 100% indicating a significant amount of heterogeneity.^[27]^

## 3. Results

The search yielded a total of 1031 articles, all of which included the search terms and met the criteria established by the research team. This filter was applied to the title and/or abstract of the articles. After this, the primary criterion of elimination of duplicates was used. Sixty articles were evaluated as a full text for inclusion in the meta-analysis and systematic review. As a result, thirty-four studies were excluded, as their primary and secondary results did not match those of this review or because they did not meet the established criteria for good data extraction. After exclusion, 26 articles were considered for analysis (N = 12,969; patients, imaging, and cadavers) (Fig. 4).^[13,28–51]^

### 3.1. Characteristics of the included studies and their population

Twenty-six studies that met the inclusion criteria were included. The total number of subjects among all included studies (N) was 12,969, with an average of 499 subjects in each study. In the studies,^[13,28]^ which accounted for 375 subjects (2.89% of the total sample), the sex of the participants was not specified. The remaining 24 studies^[29–51]^ included both males and females in their samples. These studies had in total 5802 men, representing 44.73% of the total sample, and 6792 women, representing 52.37% of the total sample (Tables 1 and 2).

**Table 1:** Summary of the articles included in this review.

Author and year	Sample characteristics	Technique	Clinical correlation
Selcuck, et al, 2006^[67]^	Patients: 330 Sex: Not specified Age: Not specified	Computed tomography (CT)	Out of 330 patients, 151 (22.8%) had at least 1 septum in the MS. Among them, 134 patients (20.3%) had an anteriorly located septum. The enlargement of the infraorbital fissure was present in 86 (64.1%) of patients that presented an anteriorly located septum, showing a significant correlation between these 2 factors. MS hypoplasia was found in 31 patients (4.6%). A significant correlation was found between MS hypoplasia and a larger orbit, where 26 patients with MS hypoplasia also presented a larger orbit (83.8%).
Butaric, et al, 2007^[18]^	Patients: 109 Sex: M: 57; F: 52 Age: Not specified (15–20 years old)	Magnetic resonance imaging (MRI)	It was observed that the size (*r*^2^ = 0.502; *P* < .001) and height (*r*^2^ = 0.589; *P* < .0001) of the MS contribute to variations in the distance between the ostium and the floor of the MS. A larger MS meant greater distances between the 2 components. Women had a smaller MS (*P* = .001) and the distance between the ostium and the floor of the MS was also smaller (*P* = .001).
Rysz and Bakoń, 2009^[64]^	Patients: 111 Sex: M: 52; F: 59 Average age not specified	Computed tomography (CT)	Through 111 CT images, 222 maxillary sinuses were studied. Among them, 29 (13%) presented infraorbital ethmoid cells (Haller cells), 49 (26%) presented at least 1 septum in the MS, and 6 (3%) had infraorbital recess in the MS. It was observed that the mean width of the nasolacrimal duct increased on the same side where infraorbital ethmoid cells were present (*P* < .01) or when the MS septum was absent (*P* < .01).
Neugebauer, et al, 2010^[49]^	Patients: 1029 Sex: M: 536; F: 493 Average age: 40.9 ± 20.03 years old (4 to 98 years old)	Cone beam computed tomography (CBCT)	Out of 1029 patients, 484 (47%) had at least 1 septum in the MS. Among them, 253 (52.3%) presented a septum in 1 of the sinuses and 141 (29.1%) presented a septum in each sinus. There was no statistical difference in the prevalence of the septum regarding the age, sex, and location of the septum.
Smith, et al, 2010^[74]^	Patients: 883 Sex: M: 385; F: 498 Average age: 44.2 years old (4–99 years old)	Cone beam computed tomography (CBCT)	Thickening of the MS mucosa was found in 50% of patients, a septum in the MS was present in 19.4% of patients, and pneumatization of the middle ethmoid cells was observed in 67.5% of patients. The pneumatization of the middle ethmoid cells was presented bilaterally in 43.2% of cases, while in 12.3% of cases it was located on the left side, and in 13.0% of cases it was located on the right side. 49.3% of patients with pneumatization of the middle ethmoid cells also had evidence of maxillary sinusitis. Despite this, no statistical relationship was observed between the pneumatization of the middle ethmoid cells and the development of maxillary sinusitis.
Gracco, et al, 2012^[32]^	Patients: 513 Sex: M: 221; F: 292 Average age not specified (12–60 years old)	Cone beam computed tomography (CBCT)	A total of 513 patients and their sinuses were studied. Among them, 206 (40.1%) presented mucosal thickening, which affected 258 sinuses (25.1%) in total. Age was a significant indicator of mucosal thickening (*P* < .001), with 41- to 60-year-old patients showing a 401% higher risk compared to 12- to 18-year-old patients. Additionally, 52 patients (10.1%) presented pseudocysts, which affected 59 sinuses (5.75%) in total. Sex was found to be a significant indicator of pseudocysts (*P* = .027), with male patients showing a 196.3% higher risk.
Lana, et al, 2012.^[43]^	Patients: 500 Sex: M: 238; F: 262 Average age: 52 years old (16 to 86 years old)	Cone beam computed tomography (CBCT)	Pneumatization of the MS was found in 416 (83.2%) patients, being the most common anatomical variation found. Additionally, 222 patients (44.4%) had a septum in the MS, 24 (4.8%) had hypoplasia of the MS, and 13 (2.6%) had exostoses.
Rege, et al, 2012^[58]^	Patients: 1113 Sex: M: 435; F: 678 Average age: 49 years old (12–85 years old)	Cone beam computed tomography (CBCT)	In 68.2% of cases, an anomaly was found. The most prevalent one was the thickening of the mucosa of the MS (66%), followed by retention cysts (10.1%), and opacification of the MS (7.8%). No relationship was found between the proximity of periapical lesions and the presence of inflammatory anomalies (*P* = .124).
Kaygusuz, et al, 2014^[38]^	Patients: 99. 65 (study group) had chronic rhinosinusitis and 34 (control) didn’t have. Sex: M: 74; F: 25 Average age: 32.2 years old	Computed tomography (CT)	None of the anatomical variations studied, including the deviation of the nasal septum, middle ethmoid cells, hyperneumatization of the ethmoid bulla, pneumatization of the uncinate process, nasal eminence, paradoxical middle turbinate, sphenoethmoidal cells (Onodi cells), and infraorbital ethmoid cells (Haller cells), showed a statistical significance when comparing the study group with the control group.
Craiu, et al, 2015^21]^	Patients: 50 Sex and Average age: M: 16; F: 34	Cone beam computed tomography (CBCT)	Pneumatization of the sphenoethmoid-maxillary cells (Sieur cells) was present on the right side in 58% of cases and on the left side in 64% of cases. Pneumatization of the maxillary recesses of the sphenoidal sinus was present on the right side in 20% of cases and on the left side in 22% of cases. Hyperneumatization was also found in the anterior wall of the pterygopalatine fossa.
Capelli and Gatti, 2016^[20]^	Patients: 70 34 patients with chronic rhinosinusitis (study group) and 36 patients without symptoms (control group) Sex: M: 34; F: 36 Average age: 46 years old	Cone beam computed tomography (CBCT)	A clear association was observed between the thickening of the MS mucosa (≥2mm) and chronic rhinosinusitis (*P* < .01). The thickening of the MS mucosa and the closure of the MS ostium were found to be associated with chronic rhinosinutysis.
Lee, et al, 2016^[44]^	Patients: 81 81 underwent MS lift Sex: M: 54; F: 27 Average age: 51.48 years old	Computed tomography (CT)	Patients with anatomical variations in the osteomeatal complex presented higher rates of postoperative complications after a MS lift surgery. A correlation between nasal septum deviation (*P* = .206), middle ethmoid cells and paradoxical middle turbinate (*P* = .276), and infraorbital ethmoid cells or Haller cells (*P* = .009) was evaluated through statistical analysis, which showed that the variation of the Haller Cells had the greatest statistical significance among the variations studied.
Nunes, et al, 2016^[50]^	Patients: 200 321 MS evaluated; 143 with at least 1 posterior maxillary tooth with periapical lesion Sex: M: 72; F: 125 Average age: 41.2 years old	Cone beam computed tomography (CBCT)	It was observed that maxillary teeth with periapical lesions had a greater frequency of anomalies in the MS, with the most frequent one being the thickening of the mucosa. It was found that a greater proximity between the periapical lesion an the MS resulted in more frequent sinus anomalies.
Shahidi, et al, 2016^[68]^	Patients:198 Sex: M: 68; F: 130 Average age: Not specified	Cone beam computed tomography (CBCT)	Out of 396 examined sinuses, 228 (57.8%) presented pneumatization of the alveolar process of the MS, 180 (45.5%) presented a septum of the MS, and 96 (24.2%) presented anterior pneumatization of the MS. In addition, the posterior superior alveolar artery was absent in 8 sinuses (7%), intraosseous in 242 sinuses (65.7%), below the MS membrane in 76 sinuses (20.6%), and in the outer cortex of the MS walls in 50 sinuses (13.5%).
Shin, et al, 2016^[72]^	Patients: 59 Sex: M: 21; F: 38 31 patients (M: 11; F: 20) had fungal ball (study group) and 28 (M: 10; F: 18) did not (control group) Average age: 56.81 ± 13.8 years old (study group), 44.39 ± 15.03 years old (control group), (22 to 74 years old)	Computed tomography (CT)	The study group had a greater number of middle ethmoid cells (61.3% vs 28.6%, *P* = .006) and infraorbital ethmoid cells (41.9% vs 30.4%), although the infraorbital ethmoid cells were not statistically significant (*P* = .348). Patients with fungal ball of MS had a narrower ethmoidal infundibulum on average (3.23 ± 0.69 vs 3.99 ± 1.17 mm, *P* < .001), but longer (9.71 ± 1.43 vs 8.23 ± 1.72 mm, *P* < .001) compared to the control group.
Arslan, et al, 2017^[7]^	Patients: 5166 Sex: M: 2692; F: 2474 Average age: 37 ± 13.5 years old (16 to 70 years old)	Computed tomography (CT)	Mucous retention cysts were present in 1429 (27.6%) of patients. Among them, 88.7% presented at least 1 naso-paranasal anomaly. A significant correlation was observed between RCM and obstruction of the osteomeatal complex, presence of accessory ostium of the MS, and abnormalities of the middle ethmoid cells (*P* = .001, *P* = .016, and *P* = .03). The sinuses with RCM were present in a greater proportion on the same side where obstruction of the osteomeatal complex, anomalies of the ethmoid cells, and ostium of the accessory sinus was present (*P* = .001, *P* = .001, *P* = .052).
Khojastepour, et al, 2017	Patients: 120 Sex: M: 63; F: 57 Average age: 27.78 ± 9.93 years old	Cone beam computed tomography (CBCT)	The presence and surface area of the infraorbital ethmoid cells (Haller Cells) showed a significant association with maxillary sinusitis. The angulation of the uncinate process and the size of the MS ostium showed no significant correlation with the development of maxillary sinusitis.
Avsever, et al, 2018^[39]^	Patients: 691 Sex: M: 423; F: 268 Average age: 45 years old (5–84 years old)	Cone beam computed tomography (CBCT)	Out of 691 patients, 548 (79.3%) presented accidental findings in the paranasal sinuses, with a total of 1109 findings, most of them located in the MS (61.23%). The most common pathology found in the MS was thickening of the MS mucosa, followed by polypoid thickening of the MS mucosa.
Baser, et al, 2019^[10]^	Patients: 54 54 patients presented unilateral antrochoanal polyp (ACP) Sex: M: 21; F: 33 Average age: 22.92 ± 13.95 years old (6 to 56 years old)	Computed tomography (CT)	The antrochoanal polyp (ACP), sides had a greater volume (17.88 ± 5.16 mm^3^) than the non-ACP sides (16.37 ± 4.55 mm^3^). Concha Bullosa was observed on the ACP side in 23 patients (42.6%), while on the non-ACP side, Concha Bullosa was present in 21 patients (38.9%). Sinuses with ACP presented Agger Nasi Cells in 47 patients (87.0%), while sinuses without ACP had it present in 42 (77.7%). Finally, on the ACP side, 14 patients (25.9%) presented hyperpneumatized ethmoid bulla, while it was observed in 12 patients (22.2%) on the non-ACP side.
Dedeoğlu and Altun, 2019^[22]^	Patients: 140 89 belonged to the adult group, differing in dentate and edentulous. 51 to the young group. M: 66; F: 74 Average age: 5.17 ± 18 years old (20–79 years old)	Cone beam computed tomography (CBCT)	In the adult group, the presence of accessory ostium of the MS (*P* = .009) and infraorbital ethmoid cells (*P* = .75) was greater than in the young group. When comparing the edentulous adult group with the dentate adult group, there were no important differences regarding the presence of MS pathologies, MS septum, and infraorbital ethmoid cells, but there was significant variation in dentate patients regarding the presence of accessory ostium of the MS (0.015)
Akay, et al, 2020^[2]^	Patients: 204 Sex: M: 97; F: 107 Average age: Not specified (18–78 years old)	Cone beam computed tomography (CBCT)	It was found that the height of the MS ostium and the maximum angle of septal deviation was significantly higher in male patients (*P* < .05). Additionally, it was observed that as the height of the MS ostium increased, so did the presence of the MS septum (*P* < .05)
Al-Zahrani, et al, 2020^[3]^	Patients:505 333 (65.9%) partially edentulous, 147 (29.1%) dentate and 25 (5%) edentulous Sex: M: 246; F: 259 Average age: Not specified (18–69 years old)	Cone beam computed tomography (CBCT)	Out of 505 patients studied, 232 (45.9%) presented one or more septa in the MS. In those patients, the septa were present in 370 sinuses (37%). In 64% of cases, the septa was located on the right side, while in 85.7% of all septa, the location was mediolateral. Among male patients 58.8%, presented a septum in the MS, while female patients presented 34% (*P* < .001).
Amine, et al, 2020^[4]^	Patients: 300 Sex: M: 117; F: 183 Average age: Not specified	Cone beam computed tomography (CBCT)	The ventilation of the MS was measured by assesing the patency of the sinus ostium, which was observed in 273 cases (91%). A septum in the MS was present in 104 patients (34.66%). Total compartmentalization of the MS was observed in 15 cases (6 %), while hypoplasia was present in 35 patients (11.6%). Aplasia was not observed (0%) and prolapse was observed in 5 patients (1.6%). A correct position of the antral alveolar artery was observed in 161 cases (53%). Mucosal thickening was present in 123 patients (41%), opacity of the sinus was observed in 12 cases (4%), and the presence of polyps and cysts was observed in 61 patients (20.33%).
Anbiaee, et al, 2020^[5]^	Patients: 199 Sex: M: 159; F: 40 Average age: M: 30.36 (±14.71) years old F: 32.65 (±13.22) years old	Computed tomography (CT)	The volume of DM was statistically higher in men than in women (*P* < .001). The volume and pneumatization of the MS showed no association with anatomical variations of the nasosinus (deviation of the nasal septum, size of the MS ostium, middle ethmoid cells and MS septum).
Aoki, et al, 2020^[6]^	Patients: 200 Sex: M: 93; F: 107 Average age: 53 years old (18–85 years old)	Cone beam computed tomography (CBCT)	Canalisis sinuosus (CS) was present in 133 cases, of which 61 (45.86%) were found unilaterally and 72 (54.14%) bilaterally. Male patients presented more CS (*P* < .05). No significant relationship was found between the presence of CS and age. Additionally, no significant relationship was found between the diameter and the end of the CS path.
Berjis, et al, 2020^[12]^	Patients: 45 cadavers Sex: Not specified Year: 18–60 years	Endoscopic nasal and paranasal dissection	Middle ethmoid cells had a typical form in 40 cases (88.9%), while in 3 cases (6.7%) they were in medial, and in 2 cases (4.4%) they were in lateral form. 40 cases (88.9%) presented nasal eminence, 17 cases (37.8%) had sphenoethmoidal cells, 13 cases (28.9%) had an accessory ostium of the MS, and 7 cases (15.6%) presented middle ethmoid cells. The location of the MS ostium was in the lower 1/3 of the lunate hiatus in 5 cases (11.1%), in 4 cases (4.4%) it was located in the upper 1/3, and in 5 cases (11.1%) it was located in the middle 1/3.

ACP = antrochoanal polyp, CBCT = cone bean computed tomography, CS = canalisis sinuosus, CT = computed tomography, MS = maxillary sinus.

**Table 2:** Description of anatomical variations reported in the included articles.

Anatomical variations	Authors and year	Country of affiliation
Haller’s cells (Infraorbital ethmoid cells)	Rysz and Bakoń, 2009	Poland
	Kaygusuz, et al, 2014	India
	Capelli and Gatti, 2016	Italy
	Lee, et al, 2016	South Korea
	Shin, et al, 2016	South Korea
	Khojastepour, et al, 2017	Iran
	Dedeoğlu and Altun, 2019	Turkey
	Akay, et al, 2020	Turkey
Onodi’s cells (Sphenoethmoidal cells)	Kaygusuz, et al, 2014	India
	Craiu, et al, 2015	Romania
	Berjis, et al, 2020	Iran
Nasal septum deviation	Kaygusuz, et al, 2014	India
	Lee, et al, 2016	South Korea
	Akay, et al, 2020	Turkey
	Anbiaee, et al, 2020	Iran
Pneumatization of anterior ethmoid cells	Kaygusuz, et al, 2014	India
	Baser, et al, 2019	Turkey
	Berjis, et al, 2020	Iran
Thickening of the MS mucosa	Smith, et al, 2010	U.S.
	Gracco, et al, 2012	Italy
	Rege, et al, 2012	Brazil
	Capelli and Gatti, 2016	Italy
	Nunes, et al, 2016	Brazil
	Avsever, et al, 2018	Turkey
	Amine, et al, 2020	Morocco
MS hypoplasia	Selcuck, et al, 2006	Turkey
	Lana, et al, 2012	Brazil
	Amine, et al, 2020	Morocco
Pneumatization of middle ethmoid cells (Bullous shell)	Smith, et al, 2010	U.S.
	Kaygusuz, et al, 2014	India
	Capelli and Gatti, 2016	Italy
	Lee, et al, 2016	South Korea
	Shin, et al, 2016	South Korea
MS accessory ostium	Capelli and Gatti, 2016	Italia
	Arslan, et al, 2017	Turkey
	Dedeoğlu and Altun, 2019	Turkey
	Berjis, et al, 2020	Iran
MS’s Septum	Selcuck, et al, 2006	Turkey
	Rysz and Bakoń, 2009	Poland
	Neugebauer, et al, 2010	Germany
	Smith, et al, 2010	U.S.
	Lana, et al, 2012	Brazil
	Poleti, et al, 2014	Brazil
	Shahidi, et al, 2016	Iran
	Dedeoğlu and Altun, 2019	Turkey
	Akay, et al, 2020	Turkey
	Al-Zahrani, et al, 2020	Saudi Arabia
	Amine, et al, 2020	Morocco
	Anbiaee, et al, 2020	Iran

MS = Maxillary sinus.

### 3.2. Prevalence and risk of bias

**Figure 8.**
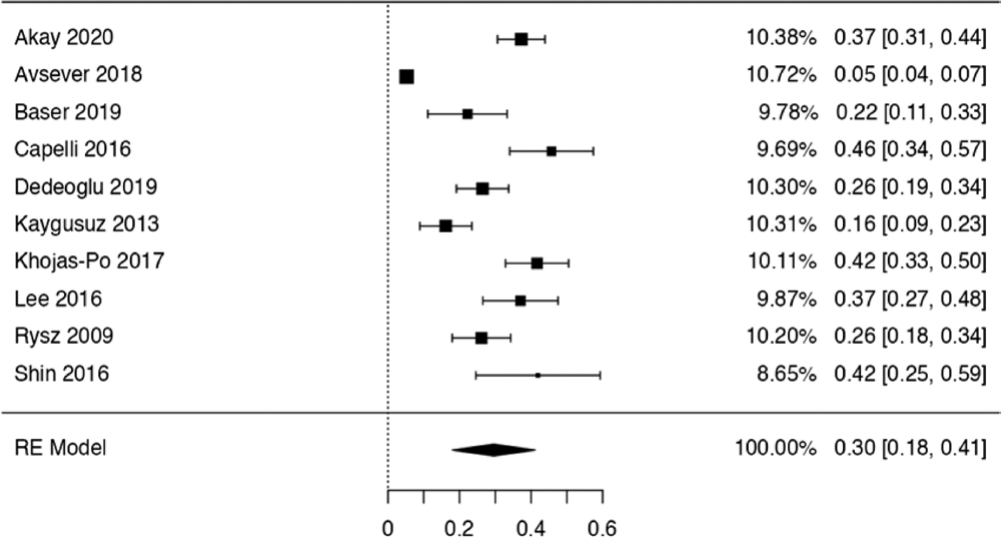
Forest plot of the prevalence variations of Haller cells. RE = random effect model.

**Figure 9.**
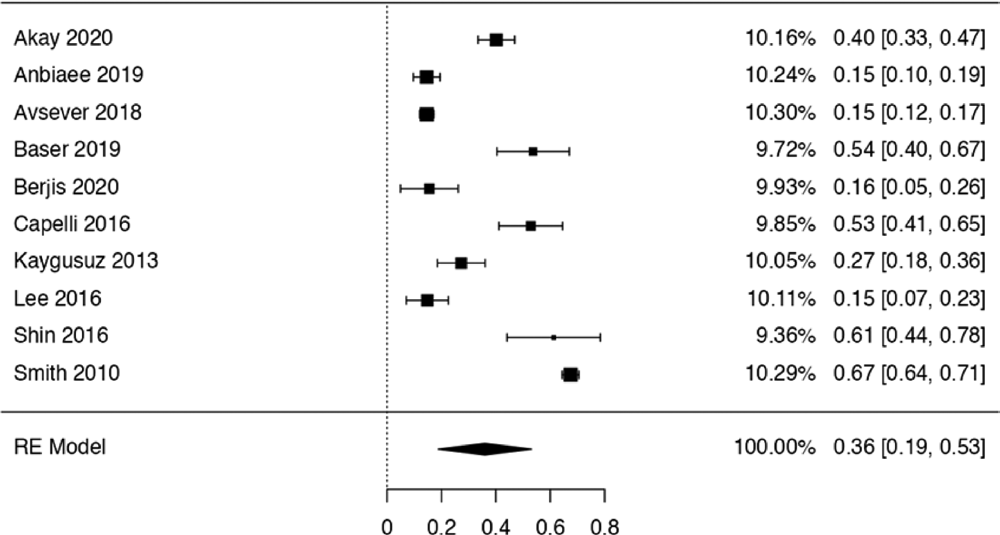
Forest plot of the prevalence concha bullosa. RE = random effect model.

**Figure 10.**
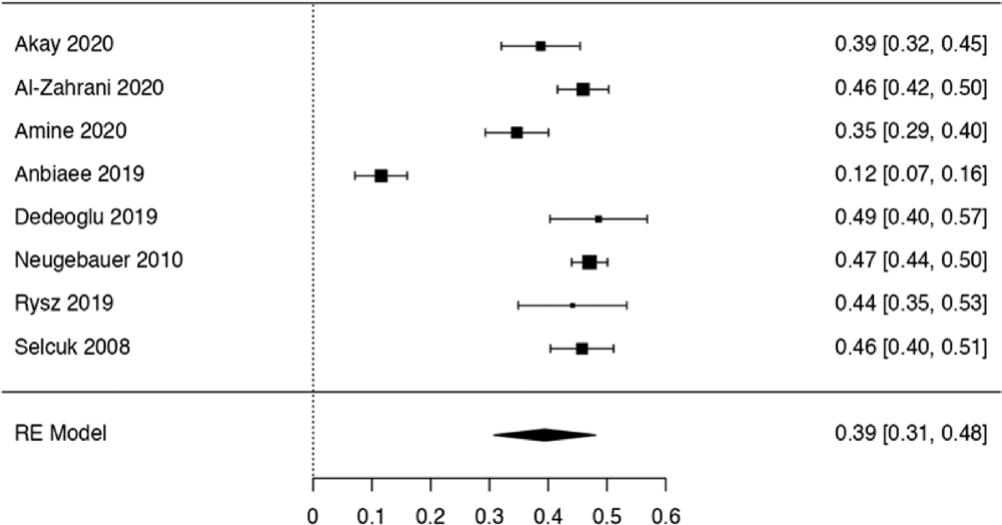
Forest plot prevalence of the number of septa. RE = random effect model.

**Figure 11.**
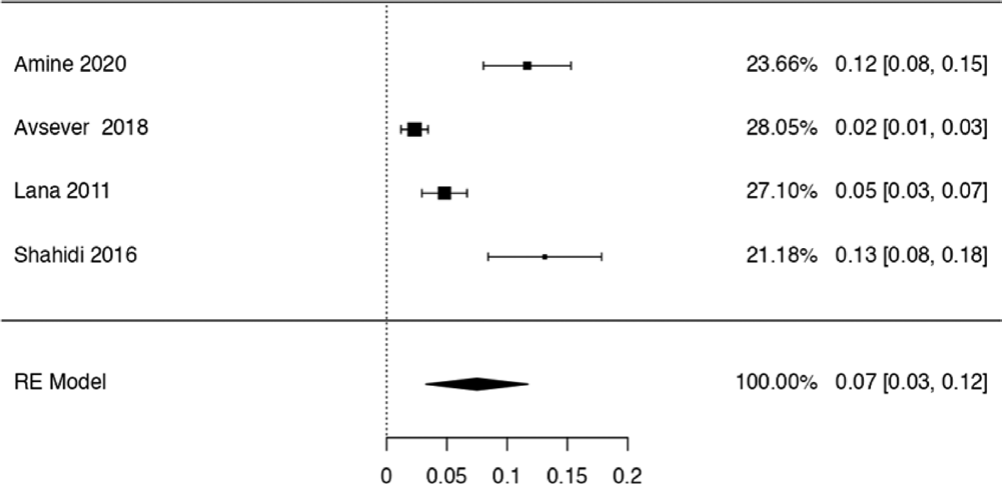
Forest plot of the hipoplasic sinus. RE = random effect model.

**Figure 12.**
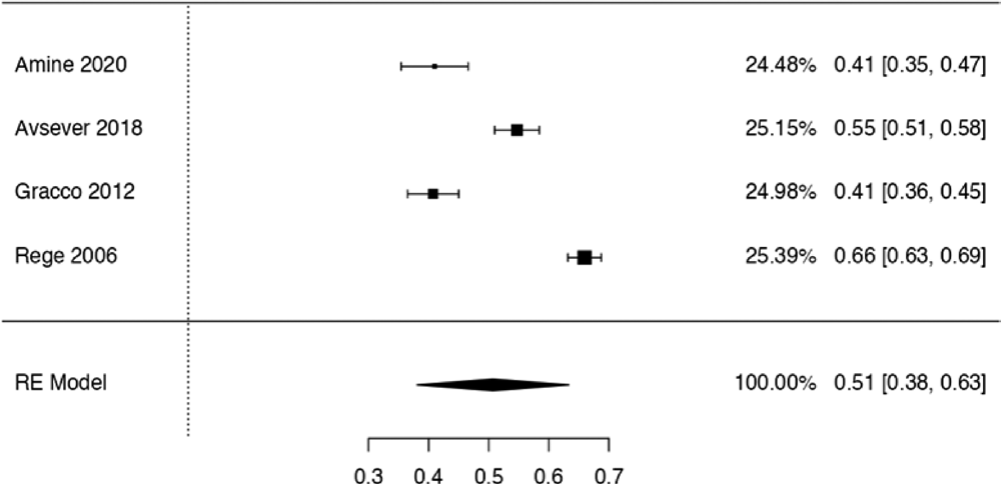
Forest plot prevalence of the thickening of mucus. RE = random effect model.

**Figure 13.**
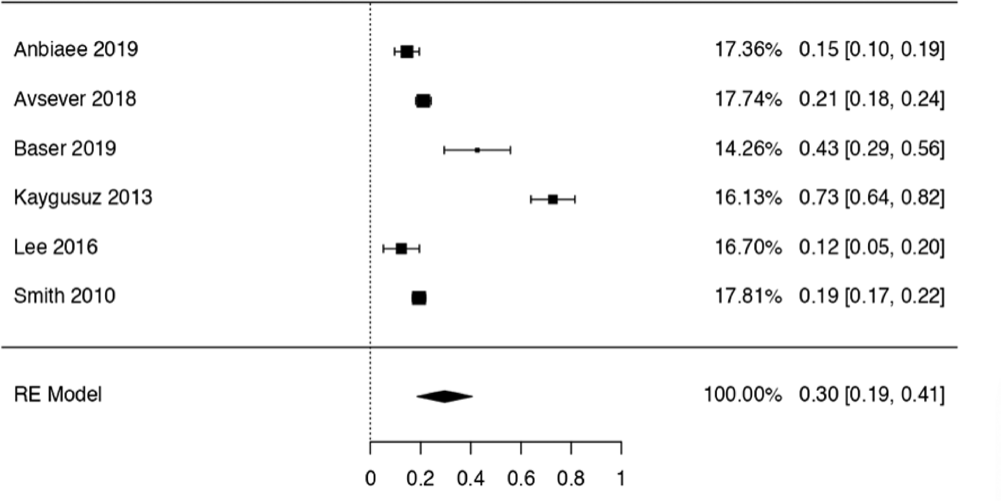
Forest plot prevalence of the nasal septum deviation. RE = random effect model.

**Figure 14.**
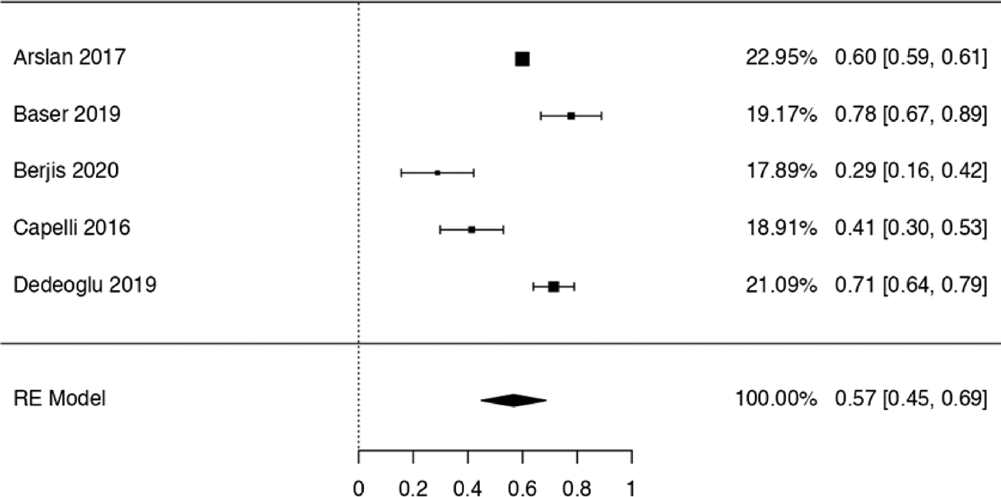
Forest plot prevalence of the ostium accessory. RE = random effect model.

**Figure 15.**
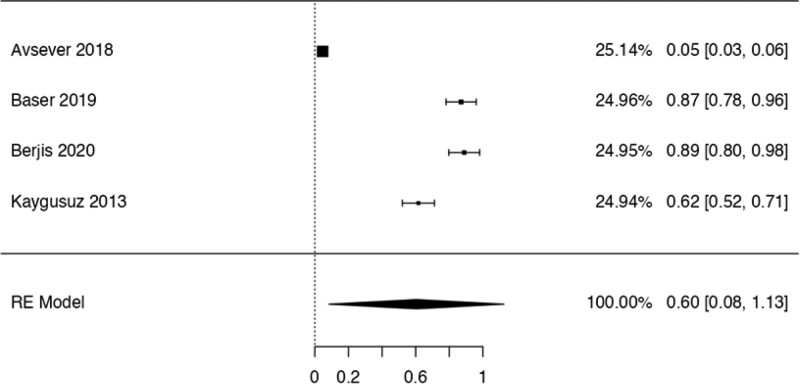
Forest plot prevalence of the variations Agger Nassi. RE = random effect model.

**Figure 16.**
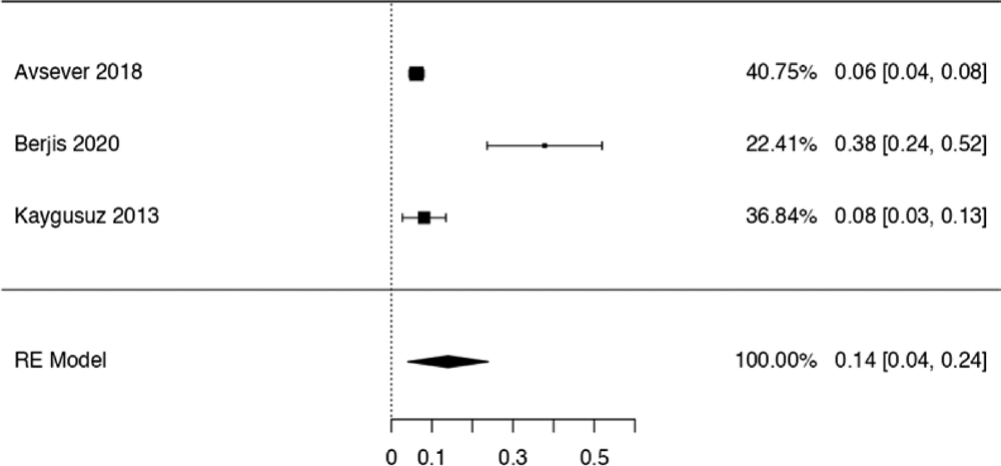
Forest plot prevalence of the celds Onodi. RE = random effect model.

This study examined the prevalence of different variants. First, 10 articles^[29,35,36,38,40,41,42,44,48,50]^ were considered to calculate the prevalence of the Haller Cell variant, resulting in a prevalence of 0.30 (0.18–0.41) (Table 3). Next, 10 studies^[28,29,32,35,36,38,41,44,50,51]^ were analyzed to determine the prevalence of the Concha Bullosa variant, resulting in a prevalence of 0.36 (0.19–0.53). Eight investigations^[13,29–32,40,45,48]^ were examined to assess the number of septa variation, showing an absolute prevalence of 0.39 (0.31–0.48). For the hypoplastic sinus variant, 4 studies^[31,35,43,49]^ were reviewed, revealing a prevalence of 0.07 (0.03–0.12). The prevalence of the mucosal thickening variant was studied considering 4 articles,^[16,31,35,47]^ which demonstrated a prevalence of 0.51 (0.38–0.63). The Nasal Septal deviation variant was analyzed using 6 investigations,^[32,35,36,41,44,51]^ resulting in a prevalence of 0.57 (0.45–0.69). The calculation of prevalence in the Agger Nasi variant was studied considering 4 articles,^[28,35,36,41]^ revealing a prevalence of 0.60 (0.08–1.13). Finally, the Onodi Cell variant was assessed using 3 investigations,^[28,35,41]^ presenting a prevalence of 0.14 (0.04–0.24). Study risk of bias was applied to 27 studies where 16 studies presented low risk of bias, while 11 studies presented high risk of bias (Table 3; Figs. 8–16).

**Table 3:** Risk of bias articles included.

Author and year	1	2	3	4	5	6	7	8	9	10	11	12	%
Akay, 2020	2	2	1	NA	2	NA	1	1	1	0	0	2	60.0
Al-Zahrani, 2020	1	0	1	NA	1	NA	1	0	0	0	0	1	25.0
Amine, 2020	1	0	1	NA	1	NA	1	0	0	0	0	1	25.0
Anbiaee, 2019	1	1	1	NA	1	NA	1	0	1	0	0	1	35.0
Aoki, 2029	1	0	1	NA	1	NA	1	0	0	0	0	1	25.0
Arslan, 2017	2	2	2	NA	2	NA	2	1	1	0	0	2	70.0
Avsever, 2018	2	1	2	NA	2	NA	1	1	1	0	0	1	55.0
Başer, 2019	1	1	1	NA	1	NA	1	0	1	0	0	1	35.0
Berjis, 2020	1	1	1	NA	1	NA	1	0	0	0	0	1	30.0
Butaric, 2007	1	2	2	NA	2	NA	1	2	1	2	1	2	80.0
Capelli, 2016	1	2	2	NA	1	NA	2	1	2	0	0	11	55.0
Craiu, 2015	2	2	2	NA	2	NA	2	2	2	0	0	2	80.0
Dedeoğlu, 2019	1	0	1	NA	1	NA	1	2	2	2	1	1	60.0
Gracco, 2012	1	0	1	NA	1	NA	1	0	2	2	2	1	55.0
Kaygusuz, 2013	2	2	2	NA	2	NA	1	1	1	0	0	2	65.0
Khojastepour, 2017	1	0	1	NA	1	NA	1	0	0	0	0	1	25.0
Lana, 2011	2	1	2	NA	2	NA	1	1	1	0	0	1	55.0
Lee, 2016	1	1	1	NA	1	NA	1	0	0	0	0	1	30.0
Neugebaer, 2010	1	2	2	NA	2	NA	1	2	1	2	1	2	80.0
Nunes, 2016	1	2	2	NA	1	NA	2	1	2	0	0	11	55.0
Rege, 2006	1	0	1	NA	1	NA	1	0	0	0	0	1	25.0
Rysz, 2009	1	0	1	NA	1	NA	1	0	0	0	0	1	25.0
Selcuk, 2008	1	1	1	NA	1	NA	1	2	1	2	0	1	55.0
Shahidi, 2016	2	2	1	NA	2	NA	1	2	1	0	0	2	65.0
Shin, 2016	2	2	2	NA	2	NA	1	1	1	0	0	2	65.0
Smith, 2010	1	0	1	NA	1	NA	1	1	2	2	2	1	60.0

Evaluation criteria – 1: exhaustive review of the literature to define the research question; 2: specific inclusion/exclusion criteria; 3: specific assumptions; 4: appropriate scope of psychometric properties (not relevant for this study); 5: sample size; 6: follow-up (not relevant for this study); 7: the authors referred to specific procedures for administration, execution and interpretation of procedures; 8: measurement techniques were standardized; 9: data were presented for each hypothesis; 10 appropriate statistics – timely estimates; 11: appropriate estimates of statistical error; 12: valid conclusions and clinical recommendations.

Score: 0 = absent; 1 = incomplete; 2 = complete; NA: not applicable.

#### 3.2.1. Anatomical variants and clinical considerations.

The included studies identified several variants, including Haller cells, Concha Bullosa, hypoplastic sinus, Agger Nasi, thickening of the MS mucosa, deviation of the Nasal Septum, Accessory ostium, and Onodi cells. The following paragraphs will describe the characteristics and findings of each variant.

The Haller Cells variant is located along the floor of the orbit, in front of the Ethmoid Bulla, and adjacent to the Ostium of the Maxillary Antrum. They originate anterior to the Ethmoid Cells in 88% of cases and posterior to them in the remaining 22%. This analysis found twelve studies^[13,29,35,36,38,40,41,42,44,48,50,52]^ mentioning this variant, with 2 reporting a clinical correlation with sinusitis.^[53]^ Other associated conditions related to this variant include nasal obstruction, frontal headache, cough, and mucocele.^[54]^

The Concha Bullosa is a variation of the Ethmoid Cells. In our study, thirteen articles^[28,29,32,33,35,36,38,40,41,44,47,50,51]^ mentioned this variant, with 2 of them reporting clinical correlations. One study related the airflow disruption, caused by this and others variants of the MS, to the formation of antrochoanal polyps.^[36]^ Another one observed a strong relationship between the presence of unilateral Concha Bullosa and a contralateral deviated septum in the development of chronic rhinosinusitis.^[55]^

The hypoplastic MS variant is rare, only 1% to 5% of normal patients with Paranasal Sinus disease presented this variation in their CT scans. In our study, 7 articles^[13,31,35,39,43,49,50]^ reported this variant, with 3 of them describing clinical correlations. A significant correlation was found between the variant and the presence of a larger orbit.^[13]^ A relation with a more medially located orbit, which increases the risk of complications during endoscopic MS surgeries, was also observed.^[56]^ Another study concluded that this variation is associated with alterations in the lateral wall of the nasal cavity, causing its approximation to the floor of the orbit.^[57]^

For the study of the Agger Nasi variant, 6 articles^[13,28,35,36,40,41]^ were reviewed, with 2 of them reporting clinical correlations. The first found no statistically significant difference in frontal sinus mucosal diseases in the presence or absence of the variant.^[58]^ Another study related airflow disruption, caused by the Agger Nasi and other variations of the MS, to the increase of formation of antrochoanal polyps.^[36]^

The thickening of the mucosa of the MS is an anomaly that results from mucus accumulation in the mucosal lining of the sinus, leading to the obstruction of the duct or gland with its epithelial lining and bone resorption due to the slow and expansive growth of this mucosa. Eight articles^[16,31,35,38,43,45,47,48]^ that reported this variant were reviewed, with 5 showing clinical correlations. The first observed a correlation between the thickening of the MS mucosa and the development of chronic rhinosinusitis.^[38]^ The second found a positive relationship between the variant and the presence of pseudocysts.^[16]^ The third found a relation between periapical lesions and the variant, while the fourth observed a weak positive relationship between age and an increase in the thickening of the mucosa of the MS.^[59,60]^ Both studies observed that periodontal bone loss was significantly associated with the thickening of the MS membrane.^[59,60]^ The last one found a significant relationship between patients with the variant and the deviation of the Nasal Septum.^[61]^

The deviation of the Nasal Septum is the torsion of the Nasal Septum that accompanies and determines the lateralization of the Nasal Pyramid. This variation is particularly relevant in people of Caucasian origin, as 80% of them present some form of this variant. The study reviewed 7 articles^[32,35,36,38,41,44,51]^ that reported this variant, where 2 showed clinical correlations. The first found a significant relationship between the presence of Nasal Septal deviation and the thickening of the MS mucosal lining.^[61]^ The second observed that the presence of said variation can limit airflow, affecting the growth of the MS.^[29]^

The accessory Ostium variant is present within 20% of patients with chronic rhinosinusitis (CRS), affecting mainly the posterior fontanel of the nasal lateral wall, which is evidenced by recirculation in up to 9% of patients. This study analyzed 7 articles^[28,29,32,34,36,38,40]^ that reported this variant, where 3 of them reported clinical correlations. The first found a significant relationship between patients that presented mucosal cysts and the presence of the accessory Ostium.^[32]^ The second observed that the presence of the variant could contribute to the development of pathologies such as Ethmoid and Maxillary sinusitis.^[62]^ The third showed that changes in the MS mucosa, such as the increase in length and area due to the presence of an accessory Ostium, seemed to increase the probability of MS pathology.^[63]^

The Cells of Onodi variation can be found in the posterior Ethmoid Cells that extend beyond the anterior wall of the Sphenoid sinus and are located between the Sphenoid Sinus and the floor of the anterior Cranial Fossa. It is capable of coming into contact with the optic nerve and internal carotid artery, exposing them to injury during surgical procedures. Five articles^[16,28,35,38,41]^ that reported this variant were analyzed and 2 reported clinical correlations. The first observed that in some cases there is a relation between the presence of the variation and the incidence of migraine appearance.^[64]^ The second reported a case of headache and acute loss of sight in a patient with retrobulbar optic neuropathy and hemianopsia in the left eye, secondary to left Sphenoethmoidal sinusitis with the presence of inflammation of the Onodi Cells.^[65]^

### 3.3. Clinical considerations for dental practice

The development of the MS, the largest paranasal cavity, begins from the 10th to 12th week of gestation, being identifiable at 16 weeks of gestation. At birth, it has a volume of 6 to 8 cubic mm, reaching the end of its development between ages 15 to 18.^[66]^ There are various publications indicating that the variations in the MS are due to alterations in the development of the cavity. These alterations affect different morphological aspects of the cavity, establishing a relationship between the presence of some variants and pathologies related to dental procedures. Some studies aim to evaluate the correlation between the placement of dental implants and the thickening of the preexisting mucosa in the MS area. However, these studies have not found a significant relationship, as other associated factors such as gender, age, and smoking could also contribute to the outcome of the implant. Nevertheless, several studies have shown a significant correlation between apical periodontitis and thickening of the sinus mucosa. Even in cases where there is severe periodontal bone loss, a higher probability of mucosal thickening could be found.^[67,68]^ Likewise, the thickening of the mucosa is an inflammatory reaction with hyperplasia of the mucosal lining of the paranasal sinus. It can be due to various causes, such as allergy, and respiratory tract infection. It is also present as a characteristic of MS hypoplasia, which may owe its origin to causes such as alteration in gestational development, among several others. This delay in development has been correlated with facial asymmetry of the upper face, being related, in turn, to the presence of asymmetry at the lower face level. This could influence a Mandibular asymmetry, and consequently alterations in the temporomandibular joint.^[57,66]^ On the other hand, there is variation in the Maxillary Sinus in terms of the presence of septa or partitions inside. Studies have reported on the correlation between these and lesions in dental surgical procedures, being related as a predictor of perforation of the membrane in the MS during the procedures of sinus floor augmentation, also being associated with a decrease in bone height of less than 3.5 mm, along with other factors, such as smoking.^[69]^

## 4. Discussion

This systematic review and meta-analysis intends to report the prevalence of various anatomical variants of the MS, elucidating their main characteristics and their relationship to pathologies of the nasal cavity and upper teeth. Studies meeting the inclusion criteria were used to perform the calculation of the prevalence of each variant, which led to the main result of this investigation: a high prevalence of most MS variants. This information is valuable as it can assist clinicians in making good clinical and diagnostic decisions in patients that presenting clinical signs associated with MS variants.

Regarding previous reviews related to MS variants, we identified 4 studies that met our inclusion criteria. In comparison to the most recent published review, there is a 3-year gap, suggesting that our study could serve as an update on different topics related to the studied variants. First, the study by Antonaya-Mira et al^[70]^ analyzed articles published between 2003 and 2008. Its objective was exclusively to examine an MS elevation procedure or technique with an osteotome, comparing it with a current technique that uses drills, where the osseointegration period was key to differentiate the treatments. Only one variation of the MS was relevant for this study, the Pneumatization of the MS, as it makes the establishment process difficult, and the mentioned techniques are used to correct it. Therefore, the study by Antonaya-Mira does not make a detailed analysis of the anatomical variants and their clinical correlation, unlike our study. On the other hand, the study by Papadopoulou^[71]^ compiled studies from 2004 to 2020, including fifty studies. However, their search items used were much broader and general than ours, since they included variations of the nasal cavity and paranasal sinuses without performing a detailed analysis of MS variants. The article by Pommer^[72]^ reviewed thirty-three investigations between 1995 and 2011 and it focused on the presence of septa in the MS, without considering other variants. Finally, the study by Vogiatzi^[73]^ analyzed variations and diseases of the MS solely through computed tomography, using twenty-two studies from 1980 to 2013.

The studies included, based on the inclusion criteria established by the research team, were evaluated for the risk of bias, in order to assess their methodological quality. In total, twenty-six studies were analyzed, of which sixteen presented moderate and low risks of bias, while 10 studies presented a high risk of bias, accounting for approximately 30% of the articles reviewed. The global results of the studies with a high risk of bias should be interpreted with caution, as many of them may have presented results that could not be interpreted or be a good guideline for clinical decision-making concerning MS variants.

Among the included studies, only primary investigations (original articles) that had a representative number of subjects in their sample were considered for the full-text analysis. As a consequence, case reports and case series were excluded from the analysis, as they did not meet the criteria described above. However, we acknowledge that in some case studies, there are special variants that are important to describe or that could be of anatomical or clinical interest, because of this it should be clarified that the articles of this type that were analyzed by the research group failed to meet the aforementioned condition.

The population included in this study, showed a fairly homogeneous distribution, with no statistically significant difference between male and female subjects. It is also worth noting that the samples used in the studies included living patients that underwent imaging studies as well as cadaveric samples of MS.

For the quantitative analysis, studies that reported the same variant were grouped and the prevalence of each variant was determined by analyzing the number of variant occurrences in relation to the total sample. The variants included were Haller cells, Concha Bullosa, Number of septa, Hypoplastic sinus, Agger Nasi, thickening of the MS mucosa, Deviation of the nasal septum, Accessory ostium, and Onodi cells. Among the mentioned, the ones that presented the greatest number of studies (between 8 and 10 studies included) were: the Haller Cells, the Concha Bullosa, and the Number of septa, where prevalence was 0.30, 0.36, 0.39 respectively. The above data shows a high likelihood of having this type of variant. It is also worth noting that many of the variants could remain asymptomatic throughout life, leading to a higher prevalence of the variants in larger or more representative populations. It should be emphasized that a high prevalence causes the results to be interpreted as a variability in the presence of this structure. Furthermore, only one variation presented a prevalence less than or equal to 0.10, which was the hypoplastic sinus where the prevalence was 0.07. Given the complexity of the normal anatomy of the maxillary sinus, there are several variants and their prevalence can be high. Therefore, multicentered studies with a higher sample size could help to understand these variants in greater detail, allowing us to understand how they influence the health of the population.

Among the most relevant clinical considerations reported in the studies reviewed was the increase in the incidence of antrochoanal polyp formation, due to the presence of the Concha Bullosa, Agger Nasi, and other anatomical variants of the MS, that caused bilateral airflow interruption. Clinical considerations involving the orbit were also found, as it was reported that the presence of hypoplasia of the MS resulted in structural changes in the orbit, changing its position and locating it more medially. This increases the risk of complications during endoscopic surgeries of the MS and could influence the position of the eyeball. The deviation of the nasal septum also showed clinical considerations, as it was found that its presence limited airflow and affected the growth of the MS. Clinical correlations regarding the Accessory ostium were observed, as its presence can contribute to the development of pathologies such as ethmoid sinusitis and maxillary sinusitis. The last variant that showed clinical correlations was the Cells of Onodi variant, which reported a case of headache and acute loss of vision in a patient with retrobulbar optic neuropathy and hemianopsia in the left eye, secondary to left sphenoethmoidal sinusitis with inflammation of the Cells of Onodi. All the above indicates that the primary clinical complications associated with variants of the MS are due to a unilateral or bilateral decrease in the space of the nasal cavity, leading to airflow obstruction or associated alterations, clinically expressed as upper airway respiratory disorders.

Our study addressed 9 anatomical variations of the MS, of which 3 showed a relationship between their presence and complications in dental procedures or the presence of dental pathologies. The study by Maska et al regarding the anatomical variation Thickening of the mucosa observed a relationship between the variation and the success in the placement of dental implants. It also found a relationship with the rate of periodontal pathologies associated with the implant. For the anatomical variation Number of Septa, the study by Iwanaga et al showed a relationship between this variation and the probability of complications due to injuries in surgical procedures. The MS Hypoplasia variant was described in 2 different studies, which established a relationship between its presence and health issues that will be described below. The first one, by Dedeoglu et al, relates the variant with the presence of other anatomical variants, such as the Haller Cells variant. It also relates the variant with the existence of associated dental pathologies. The second study, by Alsufyani et al, focused on the relationship between the hypoplastic sinus and the possibility of presenting thickening of the mucosa. It also indicates that this variation could impact the development of the face. In particular, the Hypoplasia variant shows a relationship with mandibular asymmetry, which affects the presence of pathologies of the temporomandibular joint and should be studied in depth.

## 5. Limitations

The limitations that the authors report for this review include publication bias of the included studies, since studies with different results, found in the non-indexed literature in the selected databases, may have been left out, potentially impacting our results. Other limitations include the probability of not using the most sensitive or specific search strategy for the topic studied and the potential for personal selection bias in the article filter criteria.

## 6. Conclusion

The results obtained in this review demonstrate that the MS is a complex anatomical structure for study. As a result, it is certainly complex to distinguish the presence of anatomical variations from pathological abnormalities. Therefore, knowledge of the different variations and their clinical relationships could be a useful asset for clinicians dedicated to this region. Because of this, generating new knowledge and studies on this topic could have a positive impact on interventions and treatment proposals, making them more effective and reliable. In consequence, as a research team, we believe that constant studies should be carried out to know this region in a better way.

## Acknowledgments

We gratefully thank the primary authors of the included studies.

## Author contributions

**Conceptualization:** Juan José Valenzuela-Fuenzalida, Belén Baez-Flores, Claudia Moya Medina, Macarena Cecilia Rodriguez.

**Data curation:** Juan José Valenzuela-Fuenzalida, Belén Baez-Flores, Claudia Moya Medina, Rubén Pérez, Macarena Cecilia Rodriguez.

**Formal analysis:** Juan José Valenzuela-Fuenzalida, Mathias Orellana Donoso, Macarena Cecilia Rodriguez.

**Investigation:** Roberto Ávila Sepúlveda, Javiera Leyton Silva.

**Methodology:** Belén Baez-Flores, Roberto Ávila Sepúlveda, Mathias Orellana Donoso.

**Project administration:** Belén Baez-Flores, Javiera Leyton Silva.

**Resources:** Esteban López.

**Software:** Belén Baez-Flores, Esteban López, Javiera Leyton Silva.

**Supervision:** Juan José Valenzuela-Fuenzalida, Belén Baez-Flores.

**Validation:** Macarena Cecilia Rodriguez.

**Visualization:** Juan José Valenzuela-Fuenzalida, Macarena Cecilia Rodriguez.

**Writing – original draft:** Claudia Moya Medina, Esteban López, Juan Sanchis, Mathias Orellana Donoso.

**Writing – review & editing:** Rubén Pérez, Juan Sanchis, Joe Iwanaga.

## Supplementary Material


