# Supplementary material for: Anatomical variations and abnormalities of the maxillary region and clinical implications: A systematic review and metaanalysis
Source: Medicine (Baltimore). 2023 Sep 22;102(38):e34510. doi: 10.1097/MD.0000000000034510 (PMC10519516; doi:10.1097/MD.0000000000034510)
Supplement: Supplementary file 1 [file medi-102-e34510-s001.pdf]

Supplemental Digital Content. Table S1. Searches strategies

Database	Search strategy	Results	
		15-05-23	
Medline	(((((((Maxillary sinus) OR (Maxillary bone)) AND (Variations anatomical)) OR (Morphology variations)) AND (clinical anatomy)) AND (dentistry)) OR (odontologic pathologies)) NOT (animals)	710	
SCOPUS	TITLE KEY: (((((((Maxillary sinus) OR (Maxillary bone)) AND (Variations anatomical)) OR (Morphology variations)) AND (clinical anatomy)) AND (dentistry)) OR (odontologic pathologies))	32	
Scielo	(((((((Maxillary sinus) OR (Maxillary bone)) AND (Variations anatomical)) OR (Morphology variations)) AND (clinical anatomy)) AND (dentistry)) OR (odontologic pathologies)) NOT (animals)	42	
CINHAL	(((((((Maxillary sinus) OR (Maxillary bone)) AND (Variations anatomical)) OR (Morphology variations)) AND (clinical anatomy)) AND (dentistry)) OR (odontologic pathologies)) NOT (animals)	12	
Web of science	(((((((Maxillary sinus) OR (Maxillary bone)) AND (Variations anatomical)) OR (Morphology variations)) AND (clinical anatomy)) AND (dentistry)) OR (odontologic pathologies))	101	
Google scholar	(((((((Maxillary sinus) OR (Maxillary bone)) AND (Variations anatomical)) OR (Morphology variations)) AND (clinical anatomy)) AND (dentistry)) OR (odontologic pathologies))	131	
	Total	1028	

* All searches were carried out on May 15, 2023.
